# Myxofibrosarcoma: An Unusual Occurrence in the Extremities and Its Integrated Management in India

**DOI:** 10.7759/cureus.69272

**Published:** 2024-09-12

**Authors:** Abidah Tanweer A.M., Shruthi Kamal V., Surya Rao Rao Venkata Mahipathy, Marun Raj Gunasekaran, Ragavendra N.K.

**Affiliations:** 1 Department of General Surgery, Saveetha Medical College and Hospitals, Saveetha Institute of Medical and Technical Sciences, Saveetha University, Chennai, IND; 2 Department of Plastic and Reconstructive Surgery, Saveetha Medical College and Hospitals, Saveetha Institute of Medical and Technical Sciences, Saveetha University, Chennai, IND; 3 Department of Vascular Surgery, Saveetha Medical College and Hospitals, Saveetha Institute of Medical and Technical Sciences, Saveetha University, Chennai, IND

**Keywords:** anterolateral thigh (alt) flap, chemoradiotherapy (chemo-rt), general and vascular surgeries, immunohistochemistry, spindle cell sarcoma

## Abstract

Sarcomas tend to spread to distant regions, recur locally, and exhibit various histological characteristics. They can also present differently in different areas of the body. Myxofibrosarcoma (MFS) is a typical soft-tissue sarcoma in elderly individuals, distinguished by the presence of both myxoid and fibrous tissue components. This classification might be based on its aggression, with low, middle, and high grades. We address a woman in her 50s who had spindle cell sarcoma measuring 25 x 20 cm^2^ on the anterior portion of her right thigh, along with an ulcer and discharge on top of it. The patient was evaluated with a computed tomography lower limb angiogram to look for the vasculature of the swelling. After a wide surgical excision of the mass followed by right anterolateral flap reconstruction, the patient was transferred to medical oncology for further management. The histopathological analysis revealed a grade 2 MFS. The patient has a substantial risk of morbidity and mortality due to the huge size of the sarcoma. This case report discusses the diagnosis and management of MFS, which requires integrated management involving the general surgery, vascular, and plastic surgery teams.

## Introduction

Sarcomas represent less than 1% of all adult malignancies worldwide and are a rare class of malignant tumors originating from mesenchymal tissue [1]. The World Health Organization divides the category of soft-tissue neoplasms into over 100 distinct histological subgroups according to their architectural pattern and presumed tissue of origin. Sarcomas mostly appear as a painless lump, sometimes showing signs of distant metastases, particularly in the lungs [2].

Myxofibrosarcoma (MFS) is a specific subtype of soft-tissue sarcoma (STS) that predominantly originates in the connective tissues. It is distinguished by a combination of myxoid and fibrous tissue. The condition is frequently observed in adults, particularly in older individuals, with a slight predominance in males. The incidence of MFS in India is very rare; it constitutes about 1% of all malignancies [3].

It is characterized by fibroblastic differentiation with variable myxoid stroma, pleomorphism, and a distinctively curvilinear vascular pattern [4]. The classification of MFS is determined based on the quantity of stroma, which results in the categories of low-grade, intermediate-grade, and high-grade MFS. Low-grade MFS has a higher amount of myxoid stroma, lower cellularity, and fewer mitotic figures, leading to a slower growth rate and better prognosis [5]. Intermediate-grade MFS exhibits moderate cellularity and mitotic activity with characteristics that fluctuate between low and high grades [6]. High-grade MFS is aggressive, with high cellularity and increased mitotic activity, and has a greater likelihood of metastasis [7].

MFS presents with a slow-growing swelling, often in the extremities. It may also present with ulceration or pain over the swelling [8]. On rare occasions, the patient might present with breathlessness, cough, or hemoptysis, which might be symptoms of metastasis to the lungs [2].

MFS can be diagnosed with imaging studies like computed tomography (CT) and magnetic resonance imaging to assess the size, site, and extent [9]. Its malignant nature can be diagnosed with histopathological examination. Treatment modality includes surgery like wide local excision and chemoradiotherapy. This case report elaborates on an instance of MFS in India, highlighting how the patient's symptoms significantly influenced the formulation of the management plan.

## Case presentation

**Figure 1:**
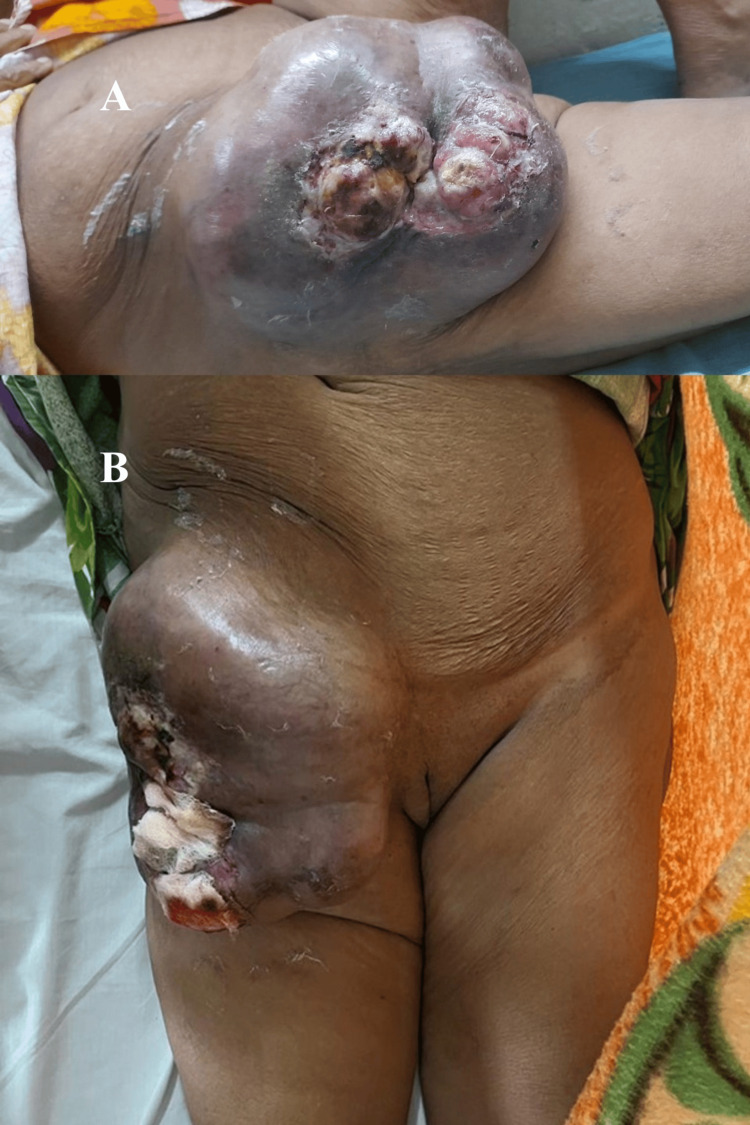
(A) Lateral and (B) anterior views of the swelling

A female patient in her 50s presented to the outpatient department with a chief complaint of swelling in her right thigh, persisting for the past year. The swelling has progressed gradually and is concomitant with an ulcer, bloody discharge, and localized pain. However, there is no reported history of restricted range of motion in the affected thigh. There was also significant history of loss of weight, approximately 20 kg in one year, associated with loss of appetite. No history of fever, bony pain, breathlessness, or hemoptysis was present. On examination, the patient was thin-built, cachexia, and pale. There was a swelling of 25 x 20 cm^2^ over the anterior aspect of the right thigh, which was an ovoid with an irregular surface, and ulceration and skin necrosis over the swelling were present, as shown in Figure 1.

The swelling felt warm and had a variable consistency with adherence to the overlying skin but was movable from the underlying structure. Bilateral inguinal lymph nodes were palpable. The tumor was thoroughly examined, and STS was suspected as a provisional diagnosis due to its prolonged presence and skin ulceration. Angiosarcoma was also suspected, as it was a very vascular swelling with feeder's vessels, though it was not a pulsatile swelling and was ruled out by a CT angiogram. Another provisional diagnosis that was made was a malignant lesion of bone involving the skin and subcutaneous tissue, which was ruled by history and imaging of the right thigh. Routine investigations were done, and the patient was found to have moderate anemia, as shown in Table 1.

**Table 1:** Blood investigations showing moderate anemia and normal coagulation profile PCV: packed cell volume; MCV: mean corpuscular volume; MCH: mean corpuscular hemoglobin; MCHC: mean corpuscular hemoglobin concentration; g: grams; dL: deciliters; fL: femtoliters; pg: picograms; %: percentage; cumm: cubic millimeter

S. no.	Test	Value	Units	Reference range
1	Hemoglobin	8.6	g/dL	12-15 g/dL
2	PCV	29.3	%	36%-46%
3	MCV	72.5	fL	83-101 fL
4	MCH	21.3	pg	27-32 pg
5	MCHC	29.4	g/dL	31.5-34.5 g/dL
6	Red cell distribution width	15.6	%	11.6%-14%
7	Total leukocyte count	10,310	cells/cumm	4,000-10,000 cells/cumm
8	Platelet count	6.46	lakhs/cumm	1.5-4.5 lakhs/cumm
9	Prothrombin time	13.5	seconds	11-13.5 seconds
10	International normalized ratio	1.18		0.8-1.1
11	Activated partial thromboplastin time	31.5	seconds	21-35 seconds

**Figure 2:**
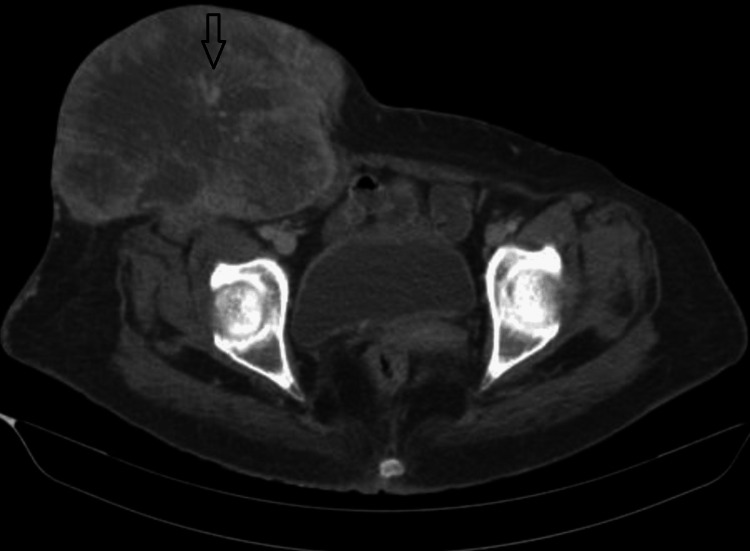
CT angiography showing the arterial supply of the tumor from the feeder vessel (black arrow) CT: computed tomography

Upon further assessment, a CT lower limb angiogram was performed on the patient, revealing indications of a probable primary malignant neoplastic lesion with the likely epicenter in the Sartorius muscle. The lesion appears to receive arterial supply from branches of the superficial femoral artery, and its venous drainage is into the great saphenous vein (Figure 2) [10].

Due to its highly vascular nature, hemoglobin was regularly monitored, and it was noted that there was a drop in the hemoglobin up to 7.3 g/dL after three days of admission, and had to undergo multiple blood transfusions. Paracetamol 650 mg twice daily orally was given for analgesia. As the patient was diabetic and hypertensive, she was on oral hypoglycemic agents like Tab. metformin+glimepiride 500 mg/1 mg twice daily and injectable insulin according to capillary blood glucose, which was monitored thrice daily with Tab. amlodipine 5 mg once daily and Tab. metoprolol 12.5 mg once daily. Culture sensitivity was done from the ulcer over the swelling, which showed the presence of Enterococcus bacteria and *Klebsiella pneumonia*, for which cefotaxime 1 g was sensitive and was administered intravenously twice daily for five days. A core needle biopsy was done, and histopathological examination showed features suggestive of spindle cell sarcoma initially as it was composed of spindle to plump oval cells with vesicular nuclei.

**Figure 3:**
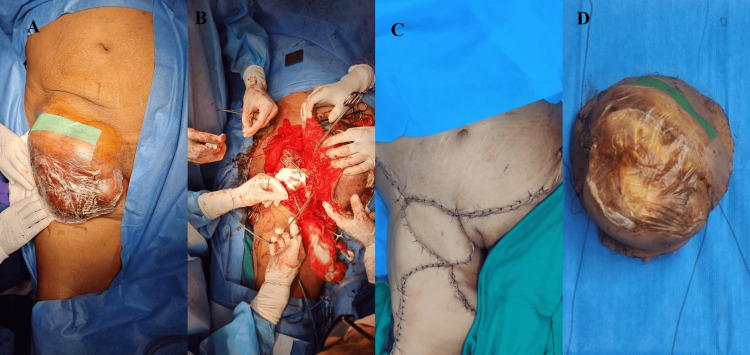
(A) Swelling is painted and draped. (B) Excision of the swelling done by the general surgery and vascular surgery teams. (C) Flap reconstruction done by the plastic surgery team. (D) The excised swelling

CT thorax showed no features of metastasis to the lungs. As the skin over the swelling was ulcerated and required immediate intervention, we proceeded with wide local excision of the tumor and then planned for adjuvant chemotherapy. The excision of the tumor was done under general anesthesia by the general surgery and the vascular surgery team. The oriented specimen was handed over to the pathologists for frozen section biopsy and their comments on the margin status. A deep margin was found to be involved. Reresection of the deep margin was done until it was negative. Anterolateral flap reconstruction was done by the plastic surgery team, as shown in Figure 3.

**Figure 4:**
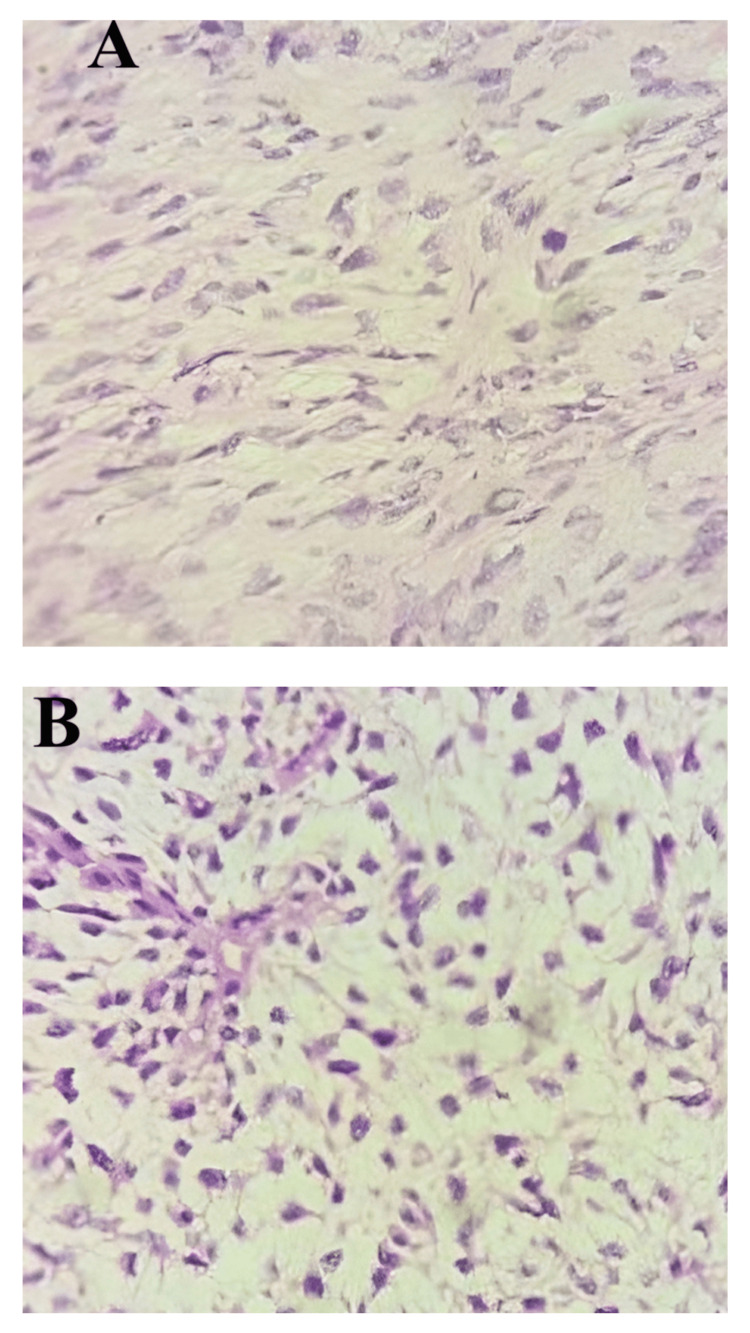
Microscopic view showing (A) myxoid stroma and (B) the curvilinear vessels with perivascular condensation

Postoperatively, she received antibiotics like cefoperazone+sulbactam 1.5 g twice daily intravenously with short-acting heparin 2500U twice daily along with analgesics like Injectable paracetamol 1 g thrice daily. Histopathological report of the specimen showed the presence of abundant myxoid stroma with the lobular pattern and elongated curvilinear vessels, which is suggestive of spindle cell sarcoma (low-grade MFS with negative margins), as depicted in Figure 4. Liposarcoma was ruled out as there were no lipoblasts, and fibromyxoid sarcoma was ruled out as there was vasculature by curvilinear vessels.

**Figure 5:**
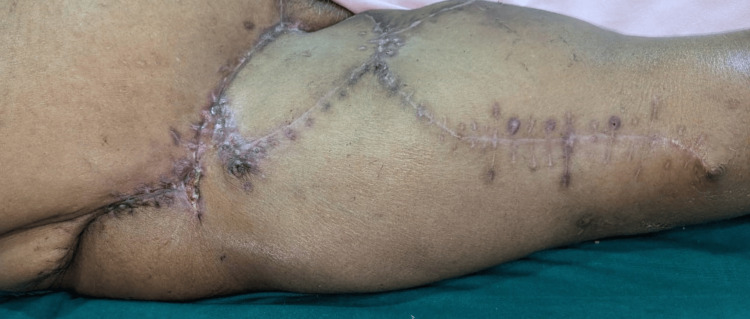
Postoperative wound showing complete flap uptake

Immunohistochemistry markers such as alpha-smooth muscle actin (SMA) tested positive in this case, confirming it as a myxoid tumor, as suggested by the pathologist. S100 was also performed in this case, yielding negative results and ruling out nerve sheath tumors and melanoma. The postoperative period was uneventful, but the uptake of the flap was good. The patient had regular follow-up appointments, with the first one scheduled two weeks after the surgery and the second at four weeks in the Plastic and Reconstructive Surgery Department. Following her six-week postsurgery period, she was referred to a medical oncologist and a radiation oncologist for further management. Subsequently, she commenced two cycles of ifosfamide 3 g, mesna 3 mg, adriamycin 60 mg, and carboplatin 60 mg for chemotherapy, followed by radiotherapy (RT). She has been disease-free after complete management, as shown in Figure 5.

## Discussion

STS is a diverse group of rare mesenchymal tumors comprising 1% of all adult malignancies [11]. Atypical fibroxanthoma, MFS, pleomorphic dermal sarcoma, myxoid melanoma, myxoid dermatofibrosarcoma protuberans, myxoid squamous cell carcinoma, and spindle cell lipoma are some of the subtypes of STS that are most common in elderly patients and affect their extremities. Although these mesenchymal tumors are more common in the lower extremities of older adults, they can also occur in the upper extremities. However, they are not as frequently observed in the head and neck, hands, and feet. Furthermore, this is an extremely rare incidence in the retroperitoneum and abdominal depth. Low-grade diseases have a low incidence of potential for metastasis and a high rate of distinct neighborhood repeats. High-grade lesions exhibit increased potential for metastasis, with the lungs being the most frequent site of metastasis. In superficially discovered lesions, the macroscopic sight is defined by many dynamically gelatinous or more firm nodules, whereas infiltrative margins are frequently observed in deep-seated illnesses [12]. In this case, the incidence of MFS in India is infrequent, and the incidence in females is even less frequent. Such cases are usually managed with neoadjuvant chemotherapy. Still, since the swelling was ulcerated and highly vascular, causing severe anemia, and was a source of sepsis for the patient, we had to do a wide local excision first, followed by adjuvant chemotherapy. Hematoxylin and eosin staining of typical MFS patients reveal curvilinear arteries, pleomorphic cells, and myxoid stroma microscopically. Fibromyxoid sarcoma is characterized by uniform fibroblast-like spindle cells with mild nuclear atypia and no significant vascularity. Therefore, it was ruled out in this case [13]. Liposarcoma contains lipoblasts, which were also ruled out. Vimentin, SMA, muscle-specific actin, and CD34 are immunohistochemical markers that rule out other differential diagnoses. S100 was primarily positive in nerve sheath tumors and melanoma. Other negative markers in MFS are desmin, CD163, CD117, and cytokeratin [14]. In cases of localized disease, radical surgery is considered the gold standard as it allows for thorough resection of the lesion, attaining free margins from tumor infiltration, and performing reconstructive procedures. In this case, the swelling was ulcerated and highly vascular, making the patient susceptible to infection and sepsis. Therefore, excision of the swelling with reconstruction was done, followed by adjuvant chemotherapy. The effectiveness of RT and CT as neoadjuvant or adjuvant treatments is still a topic of debate. In the study by Vanni et al., targeted therapy is an effective technique that enhances the results of chemotherapy [15]. MFS has a higher rate of recurrence compared to other forms of STS. The literature reports recurrence-free rates ranging from 75% to 50%. The study conducted by Tomáš et al. found that, after the surgical removal of the tumor with clear margins, a recurrence-free rate of 49.4% was reported at the five-year point [16]. The incidence of MFS in India, along with the multimodality management, makes this case stand out. It also shows that the patient's natural history is significant in making decisions in managing MFS.

## Conclusions

MFS can be rare but requires various investigations to attain the final diagnosis. It is managed by integrated teams from multiple fields. The patient's symptoms and natural history of the condition play a vital role in planning multimodality treatment. One must use the frozen section modality to prevent a recurrence and obtain a margin-free status. The histopathological study must thoroughly categorize this subtype and rule out other types of STSs. The patient must be educated about the likelihood of tumor recurrence with metastasis and the significance of regular follow-up. Further studies on MFS have to be done to understand the nature of the disease and the different treatment modalities.
